# LiDAR GEDI derived tree canopy height heterogeneity reveals patterns of biodiversity in forest ecosystems

**DOI:** 10.1016/j.ecoinf.2023.102082

**Published:** 2023-09

**Authors:** Michele Torresani, Duccio Rocchini, Alessandro Alberti, Vítězslav Moudrý, Michael Heym, Elisa Thouverai, Patrick Kacic, Enrico Tomelleri

**Affiliations:** a Free University of Bolzano/Bozen, Faculty of Agricultural, Environmental and Food Sciences, Piazza Universitá/Universitätsplatz 1, 39100 Bolzano/Bozen, Italy; b BIOME Lab, Department of Biological, Geological and Environmental Sciences, Alma Mater Studiorum University of Bologna, via Irnerio 42, 40126, Bologna, Italy; c Czech University of Life Sciences Prague, Faculty of Environmental Sciences, Department of Spatial Sciences, Kamýcka 129, Praha - Suchdol 16500, Czech Republic; d Bavarian State Institute of Forestry (LWF), Hans-Carl-von-Carlowitz-Platz-1, 85354 Freising, Germany; e Department of Remote Sensing, Institute of Geography and Geology, University of Würzburg, Würzburg, Germany

**Keywords:** GEDI, Height heterogeneity, Remote sensing, Canopy height model, Rao’s Q index, Species diversity

## Abstract

The “Height Variation Hypothesis” is an indirect approach used to estimate forest biodiversity through remote sensing data, stating that greater tree height heterogeneity (HH) measured by CHM LiDAR data indicates higher forest structure complexity and tree species diversity. This approach has traditionally been analyzed using only airborne LiDAR data, which limits its application to the availability of the dedicated flight campaigns. In this study we analyzed the relationship between tree species diversity and HH, calculated with four different heterogeneity indices using two freely available CHMs derived from the new space-borne GEDI LiDAR data. The first, with a spatial resolution of 30 m, was produced through a regression tree machine learning algorithm integrating GEDI LiDAR data and Landsat optical information. The second, with a spatial resolution of 10 m, was created using Sentinel-2 images and a deep learning convolutional neural network. We tested this approach separately in 30 forest plots situated in the northern Italian Alps, in 100 plots in the forested area of Traunstein (Germany) and successively in all the 130 plots through a cross-validation analysis. Forest density information was also included as influencing factor in a multiple regression analysis. Our results show that the GEDI CHMs can be used to assess biodiversity patterns in forest ecosystems through the estimation of the HH that is correlated to the tree species diversity. However, the results also indicate that this method is influenced by different factors including the GEDI CHMs dataset of choice and their related spatial resolution, the heterogeneity indices used to calculate the HH and the forest density. Our finding suggest that GEDI LIDAR data can be a valuable tool in the estimation of forest tree heterogeneity and related tree species diversity in forest ecosystems, which can aid in global biodiversity estimation.

## Highlights

• Monitoring forest biodiversity is crucial to prevent its decline.
• The Height Variation Hypothesis (HVH) is a key tool for biodiversity monitoring.
• Tree Height Heterogeneity assessed by GEDI data is a good proxy of species diversity.
• CHM spatial resolution, heterogeneity indices and forest density affect the HVH.
• Space-borne LiDAR GEDI derived CHMs can be used to assess forest biodiversity.

## Graphical abstract

**Figure:**
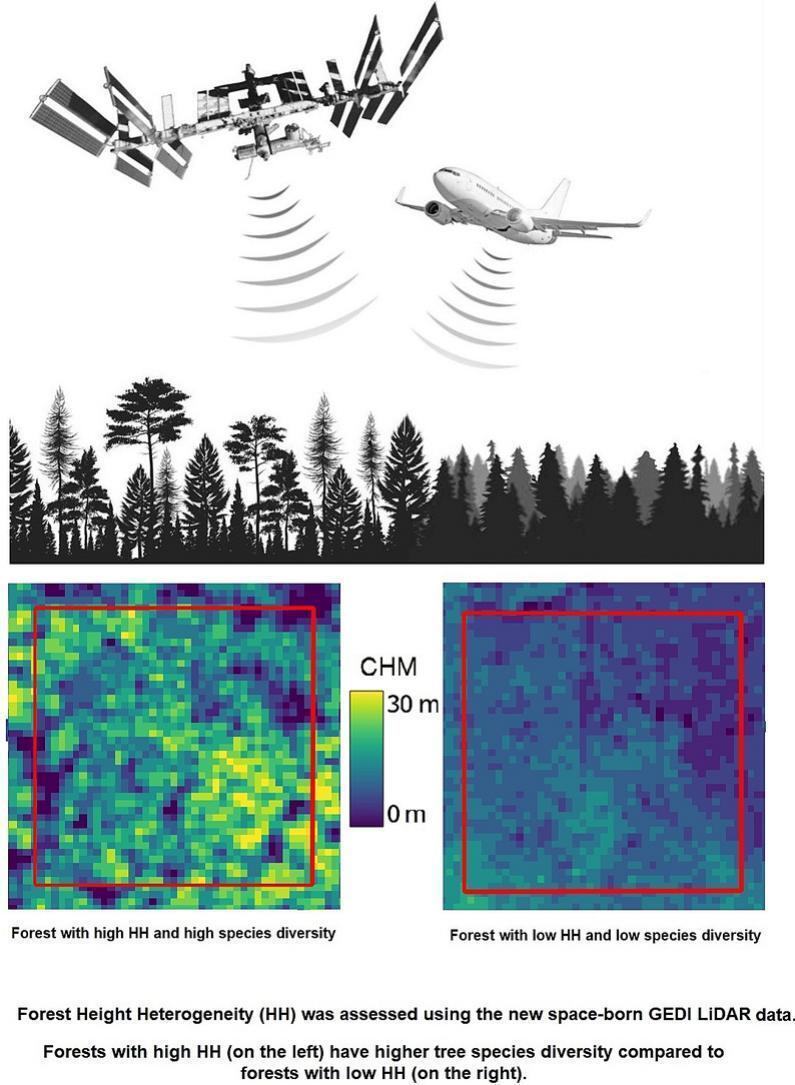

## 1 Introduction

Forests are the dominant terrestrial biome on Earth, holding most of the world’s terrestrial species (Arroyo-Rodríguez et al., 2020, Pan et al., 2013, Hansen et al., 2013, Primack et al., 2006). Most of the crucial benefits and services derived from forests including water cycle and pollution control, soil protection and carbon stock depend on the overall biodiversity condition of this ecosystem (Acharya et al., 2019). In the last 8000 years, almost 50% of the global original forest cover and the related services was lost mainly as a result of human activities; this number is likely to increase given current global deforestation rates of around 1% per year (Mittermeier et al., 1998, de Lima et al., 2020). The forest loss, the high ecosystem fragmentation together with reduction in habitat connectivity are therefore considered the major drivers of global biodiversity decline (Arroyo-Rodríguez et al., 2020, Betts et al., 2017). It is therefore important to monitor the biological diversity of forest ecosystems in order to prevent further decline and to implement significant conservation and restoration practices. Different working groups, agreements and actions as the Sustainable Development Goals (SDGs) promoted by the United Nations together with the Intergovernmental Science-Policy Platform on Biodiversity and Ecosystem Services (IPBES) or the Convention on Biological Diversity (CBD) are born in the last decades to contrast the losses of biodiversity worldwide (Skidmore et al., 2021). Other groups such as the Group on Earth observations Biodiversity Observation network (GEO BON) promote a common framework of essential biodiversity variables (EBVs) for the monitoring of biodiversity worldwide, integrating primary observations from *in situ* measurements with remote sensing data. The use of the latter data is nowadays crucial for estimating global biodiversity (Rocchini et al., 2022, Michele et al., 2018) since Earth observation represents a key tool for monitoring different habitats worldwide and their biological diversity. It became rapid and affordable to acquire large environmental information at multiple temporal and spatial scales mainly due to the fast progresses in the development of new and accurate sensors (having higher spatial and spectral resolution) and vectors (that can cover vast areas with higher revisit frequency) (Hakkenberg et al., 2018). Different remote sensing data, approaches and methodologies have been developed in the last years to estimate various aspects of biodiversity (White et al., 2010). Recent approaches aim to analyse an indirect relationship between the environmental heterogeneity, measured as the variation of the remotely sensed data and field-based biodiversity, as reviewed in Kacic and Kuenzer (2022). The Spectral Variation Hypothesis (SVH), proposed by Palmer et al. (2002) represents a perfect example. This concept, discussed and analyzed in different studies (Rocchini et al., 2004, Rocchini et al., 2022, Tagliabue et al., 2020, Rocchini et al., 2021, Torresani et al., 2021, Marzialetti et al., 2020, Sun et al., 2021, Gholizadeh et al., 2018), hypothesizes that areas with high variability of spectral information are characterized by a higher environmental heterogeneity, higher number of niches where more species can survive (Rocchini et al., 2022). Different studies showed that this approach holds true in a range of ecosystems and it is influenced by a series of factors, including the spatial resolution of the optical data (Michele et al., 2018, Rocchini, 2007), the heterogeneity indices used to estimate the variability of the optical data (e.g. the Rao’s Q index, the Coefficient of Variation, the Convex hull index) (Gholizadeh et al., 2018), the seasonality (Torresani et al., 2019) and the indices used to assess the field species diversity (e.g. Shannon’s H, species richness) (Oldeland et al., 2010).

Recent studies (Torresani et al., 2020, Tamburlin et al., 2021) have proposed to test the theory behind the SVH using LiDAR data in order to understand whether the heterogeneity of LiDAR information, used in particular to assess the Height Heterogeneity (HH) in forest ecosystems, can be used as a proxy of trees species diversity. This approach (called “Height Variation Hypothesis” -HVH) states that, the higher the forest vertical structure complexity, and hence the higher number of sub-habitats and niches that can be found in the forest and the higher the diversity of growing trees (Torresani et al., 2020, Moudrỳ et al., 2023, Moudrỳ et al., 2021). As for the SVH, this approach is influenced by different factors such as the spatial resolution of LiDAR data, the canopy cover and density of the forest, the heterogeneity indices and the LiDAR metrics used to assess the HH. To date, the relationship between HH and tree species diversity has been tested using only airborne LiDAR data (in particular using the Canopy Height Models - CHM - that according to different studies (Torresani et al., 2020, Tamburlin et al., 2021) is the most appropriate for this purpose), which makes its application limited to the availability of the dedicated flight campaigns.

In December 2018, the Global Ecosystem Dynamics Investigation (GEDI), a spaceborne LiDAR sensor from NASA onboard the International Space Station (ISS) was launched in order to produce high resolution 3D observations of the Earth’s forests (Dubayah et al., 2020, Potapov et al., 2021). GEDI data are collected from a full waveform LiDAR sensor (with 25 m diameter footprints) (Roy et al., 2021), and can be used as valuable estimates of forest structure, its heterogeneity and related biodiversity. The novel data-sets of GEDI have been used to cover information about forest canopy height (Gupta and Sharma, 2022, Marselis et al., 2022), growth dynamics (Guerra-Hernández and Pascual, 2021), vertical foliage complexity (Kacic et al., 2021), above-ground biomass (Duncanson et al., 2020, Saarela et al., 2022, Dubayah et al., 2022), biomass density (Duncanson et al., 2022), forest fuels classification (Hoffrén et al., 2023) and surface elevation (Quirós et al., 2021) which are key components for a global monitoring of forest ecosystems. Limitations at local scale arise because of the generalized footprint and the sparse sampling design (Liu et al., 2022). To extrapolate GEDI samples for continuous information on vegetation structure, multiple approaches have been developed fusing GEDI samples with passive optical images in machine learning models (Liu et al., 2022, Rishmawi et al., 2021, Kacic et al., 2021). At global scale, Potapov et al. (2021) made use of machine learning algorithms to compute and derive a global forest canopy height map (hereafter “Potapov30m”) at 30 m spatial resolution fusing GEDI and phenology metrics based on Landsat 8 OLI imagery. More recently Lang et al., 2022, Lang et al., 2022, produced a high-resolution CHM of the Earth (hereafter “Lang10m”) at 10 m resolution using Sentinel-2 optical images and a deep learning approach (convolutional neural networks).

**Fig. 1:**
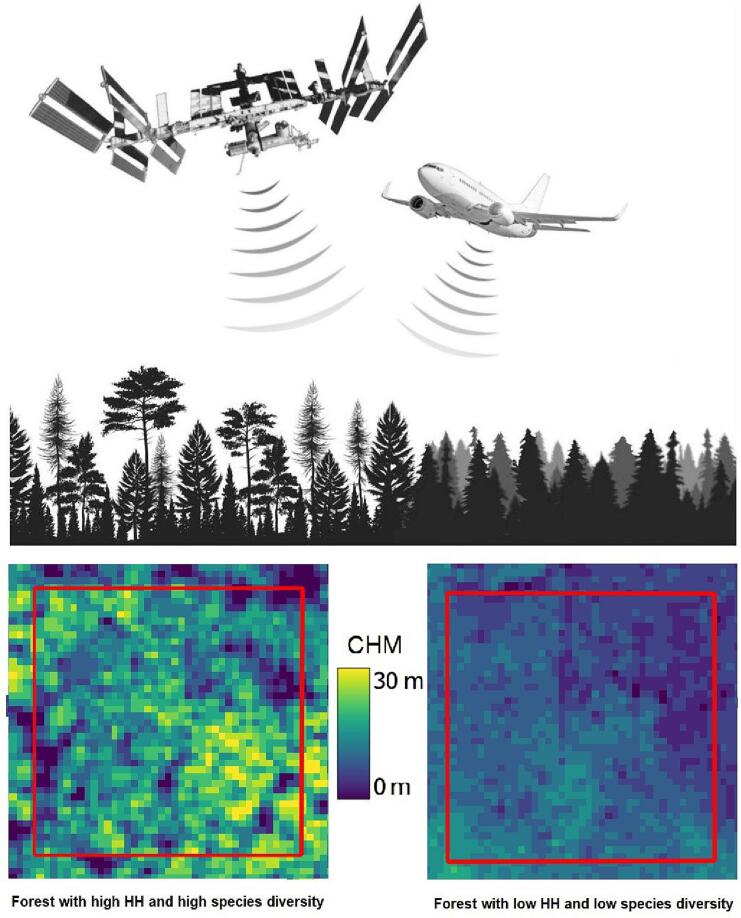
The figure summarizes the aim of our study. Forests with high HH (assessed through CHM LiDAR data) have a complex vertical structure (seen from the side in the upper figure and from above in the lower figure), high environmental heterogeneity and high tree species diversity (forest on the left). On the other hand, forest with low HH might have lower species diversity (forest on the right). This concept has been already tested measuring the HH with airborne LiDAR data. In this study we test it using the recently published and freely available LiDAR GEDI CHMs Lang10m and Potapov30m.

The aim of this study (Fig. 1) is to understand if the recently published and freely available LiDAR GEDI CHMs Lang10m and Potapov30m can be used to assess biodiversity patterns in forest ecosystems. In particular, (1) we evaluated the accuracy of both the global GEDI CHMs using ALS CHMs, (2) we assessed the relationship between *in situ* tree species diversity (using Shannon’s H index and species richness) and HH calculated using the GEDI CHMs through four heterogeneity indices (Rao’s Q, CV, Shannon’s H and Simpson’s D index); (3) we tested the effect of forest density (through the canopy cover and the number of trees) on this relationship; and (4) we proposed a visual spatialization of the best outcomes to better understand the proposed approach and the results. We tested this separately in 30 forest plots situated in the northern Italian Alps, in 100 plots in the forested area of Traunstein (Germany) and successively in all the 130 plots through a cross-validation analysis.

## 2 Material and methods

### 2.1 Study areas

**Fig. 2:**
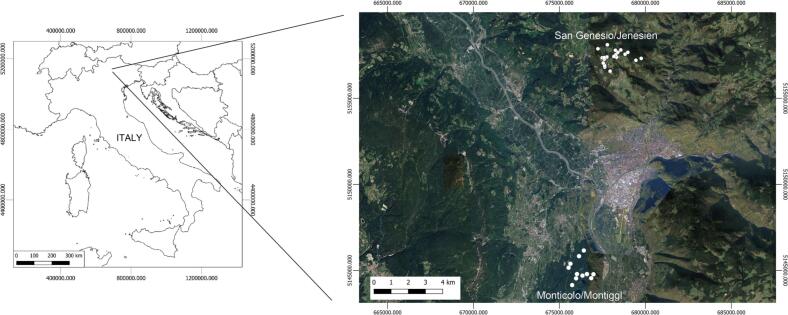
In white, the center of the 30 plots situated in the 2 forested areas of San Genesio/Jenesien and Monticolo/Montiggl in South Tyrol (Italy). Background image used: Google Image at February 21th 2023. Coordinates in WGS 84/ UTM zone 32 N (EPSG:32632).

Our approach was tested in two separate forest areas, one in Italy and one in Germany. In Italy, the approach was tested in 30 plots (with a size of 1 ha, 100 m x 100 m), randomly chosen in 2 separate forests in the Province of Bolzano/Bozen (Italy) (Fig. 2). 20 plots are located on the Salten/Salten plateau (1100 m a.s.l.), in a topographically homogeneous coniferous forest area above the municipality of San Genesio/Jenesien. Between June and August 2017, an exhaustive field data collection was performed where all the trees with a diameter at breast height (DBH) greater then 5 cm were measured and classified by species. *Pinus sylvestris*, followed by *Larix decidua* and *Picea abies* were the dominating species (95%). 5% were deciduous trees such as *Betula alba*, *Corylus avellana*, *Salix caprea* and *Sorbus aucuparia*. See Torresani et al. (2021) for further information about the area. 10 other plots have been randomly selected within a temperate forest at 490 m a.s.l. near the Monticolo/Montiggl lake in a topographically homogeneous area in the municipality of Appiano sulla Strada del Vino/Eppan an der Weinstraße. Also in this area, a field campaign conducted in Spring 2019 was carried out in order to classify species of all trees with a DBH of at least 5 cm. 51% of the measured trees were conifers, with *Pinus sylvestris* as dominant species, followed by *Larix decidua* and *Picea abies*. The remaining 49% were broad-leaves with *Castanea sativa* and *Quercus pubescens* as dominant species followed by *Populus tremula* and *Betula alba*. To obtain the exact position of each of the 30 plots, their centers and corners were geo-referenced with a GPS device (spatial accuracy ±3 m). To simplify, henceforth we will use “Italian study area” to refer to all 30 plots.

**Fig. 3:**
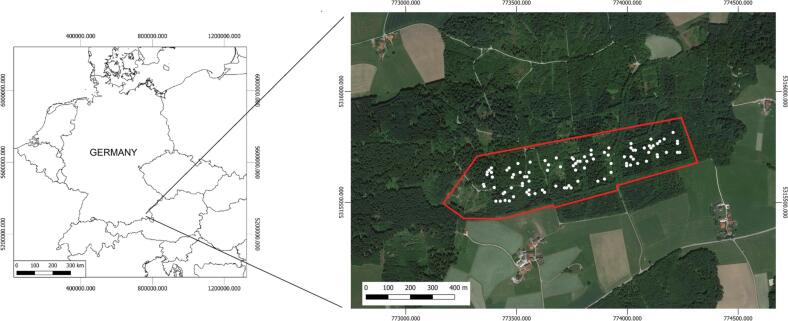
The study area Traunstein (Germany). The border of the study area is shown in red. The white dots show the center of all the plots. Background image used: Google Image at February 21th 2023. Coordinates in WGS 84/ UTM zone 32 N (EPSG:32632).

The German study area is located in the forested area of Traunstein (Fig. 3) located in the municipality of Traunstein (Germany). The area (N47°52’ E12°38’) is topographically homogeneous and it has a size of 25 ha; it is included in the ForestGEO network (https://forestgeo.si.edu/) created and censused in 2015. Within the forest, all the trees with a DBH ⩾5cm were geo-located and classified by species. 52% of trees were conifers (*Picea abies in primis*) followed by 48% of broadleaves dominated by *Acer pseudoplatanus*. Within the area, 100 plots of 1 ha of size (100 m x 100 m) were randomly chosen. As explained in a previous study (Torresani et al., 2020) due to their size, some plots might have a partially shared/overlapped area. Since the analysis is a “per plot-based analysis” the overlapping should not create statistical issues (Torresani et al., 2020).

### 2.2 *In-situ* species diversity

The *in situ* tree species diversity was calculated for each plot of both study sites, using 2 different indices: species richness and Shannon’s *H*. Species richness represents the number of different species found in each plot. Shannon’s *H* (formula 1) is one of the most famous index used in ecology for assessing alpha diversity, it is based on the abundance of each species in a certain area reflecting the evenness of the population (Shannon, 1948).

(1) $$H=-\overset{q}{\underset{i=1}{\sum }}p_{i}*\log(p_{i})$$

where:

*H* = Shannon’s H index
*q* = number of actual species
$p_{i}$ = ratio between the number of individuals for a defined species *i* and the total number of individuals within each plot.

### 2.3 LiDAR data

#### 2.3.1 GEDI LiDAR data

We estimated the HH using the recently published and freely available LiDAR GEDI CHMs Lang10m (Lang et al., 2022, Lang et al., 2022) (downloaded here: https://langnico.github.io/globalcanopyheight/) and Potapov30m (Potapov et al., 2021) (downloaded here: https://glad.umd.edu/dataset/gedi/).

Lang10m was derived fusing the GEDI and Sentinel-2 images through a deep convolutional neural network (Lang et al., 2022). It has spatial resolution of 10 m and is valid for the year 2020. The canopy top height was defined as the relative height at which 98% of the energy was returned (RH98). For the modelling GEDI observations for which the image was cloudy or snow-covered were excluded.

Potapov30m was derived using GEDI, Landsat and SRTM data and a regression tree algorithm. It has spatial resolution of 30 m and is valid for the year 2019. The estimate of GEDI canopy height used in the models represents the 95th percentile of energy return height relative to the ground (RH95). To select the highest quality training and validation data they used for the modelling only observations collected in power beam mode, during the night, and with beam sensitivity 0.9. In addition, they excluded observation collected during the leaf-off season in temperate and boreal forests.

Validation of Potapov30m using ALS data performed by the authors themselves showed ME of −3.8 m, RMSE of 9.07 m, and MAE of 6.36 m for their map (Potapov et al., 2021). Lang et al. (2022) reported similar results ME of 0.2 m, RMSE of 8.8 m, and MAE of 6.9 m for validation of their map using ALS data. In addition, Lang et al. (2022) validated the Potapov30m map and according to their results, it appeared to be less accurate than their map (ME = −4.8, RMSE  = 9.6 m; MAE  = 7.4 m).

#### 2.3.2 Local ALS LiDAR data

In order to validate the GEDI CHMs and to calculate the canopy cover we used local Airborne Laser Scanning (ALS) LiDAR data. For the Italian study area, we derived the CHM from an ALS campaign completed in 2006 by the Province of Bolzano/Bozen (free available here: http://geocatalogo.retecivica.bz.it/geokatalog/). For the German study area were used the LiDAR data derived from an ALS campaign carried out in 2010 (for the assessment of the DTM) and 2018 (for the assesment of DSM). For both study sites, the CHMs, calcuated as the difference between the DSM (derived from the point cloud using the R packege *“lidR”* through the function “rasterize canopy” with the “p2r” algorithm) and DTM (available with the point cloud for both study sites) were derived with a spatial resolution of 2.5 m (the highest possible for the Italian study area and for this reason used also for the German site) for the assessment of the canopy cover and of 10 m and 30 m for the validation of the GEDI CHMs (Lang10m and Potapov30m respectively). We refer to (Torresani et al., 2020) for more detailed information of the local ALS campaign.

### 2.4 Canopy cover and forest density

Following the work of Torresani et al. (2020) the canopy cover was calculated for each plot through the following formula:

(2) $$\mathrm{CC}=\frac{{\mathrm{px}}_{2m}}{{\mathrm{px}}_{\mathrm{tot}}}*100$$

where:

CC  = Canopy cover
${\text{px}}_{2m}$ = number of pixels with a CHM  > 2 m
${\text{px}}_{\mathrm{tot}}$ = total number of pixels

Since the point cloud of the Italian ALS data allowed to create a CHM with the highest spatial resolution of 2.5 m, this resolution was chosen to calculate the canopy cover for both Italian and German study areas. The forest tree density was estimated as the number of trees per plot.

### 2.5 Heterogeneity indices

HH was calculated using the 2 GEDI CHMs with 4 different heterogeneity indices: the Rao’s Q index, the Coefficient of Variation (CV), the Shannon’s H index and the Simpson’s D index. The Rao’s Q index (formula 3) was developed by Rao (1982), successively Botta-Dukát (2005) suggested it as a functional diversity index in ecology. Rocchini et al. (2017) proposed this measure as heterogeneity index to be used with remote sensing data using the following formula:

(3) $$Q=\overset{N}{\underset{i,j=1}{\sum }}d_{\mathrm{ij}}\times p_{i}\times p_{j}$$

where:

Q  = Rao’s Q index, used in remote sensing application
$p_{i}$*=*$p_{j}$*=*1/N = relative abundance of pixel i, j in a selected area (i.e. region of interest, raster) composed of N pixels
$d_{\mathrm{ij}}$ = distance/dissimilarity between pixel i and j ($d_{\mathrm{ij}}$ = $d_{\mathrm{ji}}$ and $d_{\mathrm{ii}}=0$)

In this study we calculated $d_{\mathrm{ij}}$ as a simple Euclidean distance based on a single layer (GEDI CHMs raster Lang10m and Potapov30m).

The CV (formula 4) largely used in various ecological researches as heterogeneity index (Gholizadeh et al., 2018, Levin et al., 2007), is calculated as follow:

(4) $$\mathrm{CV}=\mathrm{SD}/\overset{‾}{x}$$

where:

CV = Coefficient of Variation
SD = Standard Deviation of the pixel values within a selected area
$\overset{‾}{x}$ = mean of the pixel values within a selected area

The Shannon’s H index, largely used in ecology, can be used also in remote sensing application (Rocchini et al., 2017) using the following formula:

(5) $$H_{\text{rs}}=-\overset{q}{\underset{i=1}{\sum }}p_{i}*\log(p_{i})$$

where:

${\text{H}}_{\mathrm{rs}}$ = Shannon’s H index used in remote sensing
*q* = unique numerical pixel values within a selected area
$p_{i}$ = relative abundance of each q

The Simpson’s D’s index is another measure used in ecology for assessing diversity (Kumar et al., 2022, DeJong, 1975), it can be used as heterogeneity measure with remote sensing data (formula 6), relying only on the relative abundance of the pixels within the considered plot/area (Rocchini et al., 2021).

(6) $$D=\overset{n}{\underset{i=1}{\sum }}p_{i}^{2}$$

where:

*D* = Simpson index
*n* = total number of pixels of a specific value
$p_{i}$ = relative abundance of a pixel value in a CHM raster plot

### 2.6 Workflow

**Fig. 4:**
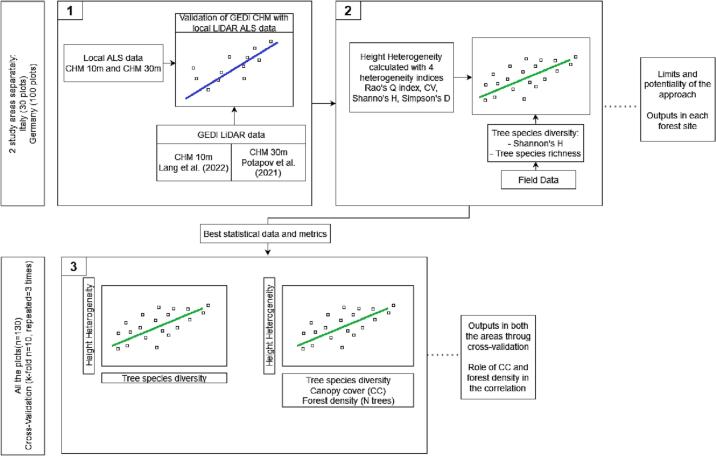
The image shows the workflow of the proposed approach.

The approach proposed in this study is summarized in Fig. 4. Firstly (point 1), in each study area (30 Italian plots and 100 German plots) we validated both the GEDI CHMs (Lang10m and Potapov30m) with local ALS LiDAR data. Successively (point 2), for each study area, we used the 4 heterogeneity indices (Rao’s Q, CV, Shannon’s H and Simpson’s D) to calculate the HH using both the GEDI CHMs (Lang10m and Potapov30m). The HHs have been successively correlated by linear regression with the *in situ* tree species diversity (assessed through the Shannon’s *H* index and species richness). The coefficient of determination ($R^{2}$) was used to estimate the fitness of the model while *P value* to measure its statistical significance. Thereafter (point 3), we tested the above mentioned correlation in all the plots (n = 130) through a cross-validation analysis (k-fold n = 10, repeated n = 3, function *“repeatedcv”*, R package “*caret*”) using the parameters that showed the highest goodness of fit ($R^{2}$) and lower root mean square error (RMSE) and mean absolute error (MAE) at the point 2. The cross-validation was tested with a single linear regression (HH vs tree species diversity indices) and with a multiple linear regression (HH vs species diversity  + canopy cover  + forest density -number of trees-). Finally, we visually show our best results in order to have a clear comparison between the best-performing GEDI CHM, the calculated HH (mapped using the *rasterdiv* R package Rocchini et al., 2021), and the tree species diversity.

### 2.7 Statistical analysis

The accuracy of both the GEDI CHMs was exterminated by comparing them with the local ALS CHMs. The Coefficient of determination ($R^{2}$) derived from the linear regression of both the variables, the RMSE and the MAE were derived as follow:

(7) $$\mathrm{RMSE}=\sqrt{1/n\overset{n}{\underset{k=1}{\sum }}{\left(X-Y\right)}^{2}}$$

(8) $$\mathrm{MAE}=1/n\overset{n}{\underset{k=1}{\sum }}|X-Y|$$

where: X is the GEDI CHM, Y is the local CHM and n is the number of pixels. $R^{2}$, RMSE and MAE were calculated also when the HH (calculated with the GEDI CHMs using the 4 heterogeneity indices) was correlated by linear regression with the tree species diversity. In this case X is the HH values, Y is the species diversity (values of Shannon’s H or species richness) and n is the number of plots.

## 3 Results

### 3.1 Validation of the GEDI CHMs

**Fig. 5:**
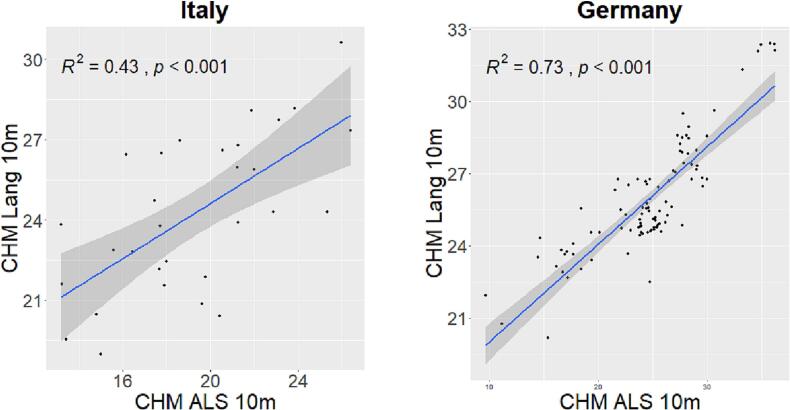
Validation of the GEDI CHM Lang10m with local ALS CHM LiDAR data at 10 m spatial resolution.

The validation of the GEDI CHM Lang10m with local ALS CHM LiDAR data at 10 m spatial resolution is shown in Fig. 5. In the 30 Italian plots the goodness of fit between the two variables reach a value of 0.43 while for the 100 German plots a value of 0.73. Both the correlations are significant (p value  < 0.05).

**Fig. 6:**
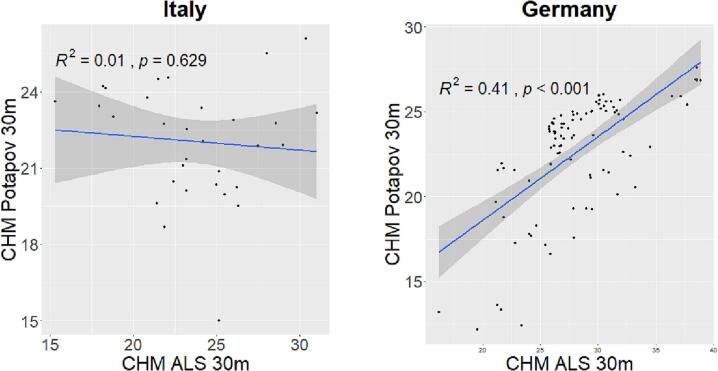
Validation of the GEDI CHM Potapov30m with local ALS CHM LiDAR data at 30 m spatial resolution.

The validation of the GEDI CHM Potapov30m with local ALS CHM LiDAR data at 30 m spatial resolution is shown in Fig. 6. In this case, the correlations show lower $R^{2}$ values. In the Italian study area the correlation is not significant with a $R^{2}$ that does not explain the variance in the GEDI CHM Potapov30m. In the German study area the correlation is significant with goodness of fit of 0.41.

$R^{2}$ values, Root mean square error (RMSE) and Mean Absolute Error (MAE) of the above mentioned correlations are shown in Table 1. Both GEDI CHM products, in relation to ALS CHM, overestimate canopy height by few meters, with a RMSE that range from 3.4 m to 6.5 m.

**Table 1:** $R^{2}$, RMSE and MAE of the correlation between local ALS CHM and CHMs derived from GEDI.

	Italy		Germany	
	Lang10m	Potapov30m	Lang10m	Potapov30m
$R^{2}$	0.43	0.01	0.73	0.41
*RMSE* (m)	5.71	4.81	3.43	6.54
*MAE* (m)	5.03	4.19	2.38	5.63

### 3.2 Canopy cover and forest density

For each of the study area, the mean of canopy cover and mean of number of trees for all plots are shown in Table 2. In the Italian study area, both the means are higher than in the German study area highlighting its higher forest density.

**Table 2:** Mean of canopy cover and number of trees for all plots in each study area.

	**Canopy Cover - canopy cover -**	**Number of trees/plot (1** **ha)**
**Italy**	97.19	995.23
**Germany**	92.14	656.31

### 3.3 Correlations between the HH and the tree species diversity

**Fig. 7:**
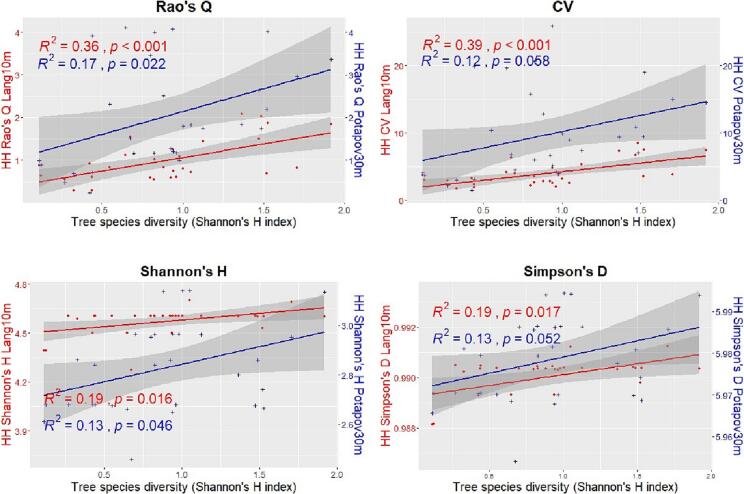
Linear regressions between the tree species diversity estimated through the Shannon’s H index and the HH calculated with the 4 heterogeneity indices (Rao’s Q, CV, Shannon’s H and Simpson’s D) using both the GEDI CHMs Potapov30m (blue points and line) and Lang10m (red points and line) in the Italian study area. In order to better compare the results, in the sub-plots of Shannon’s H and the Simpson’s D, 2 different y scales have been used.

**Fig. 8:**
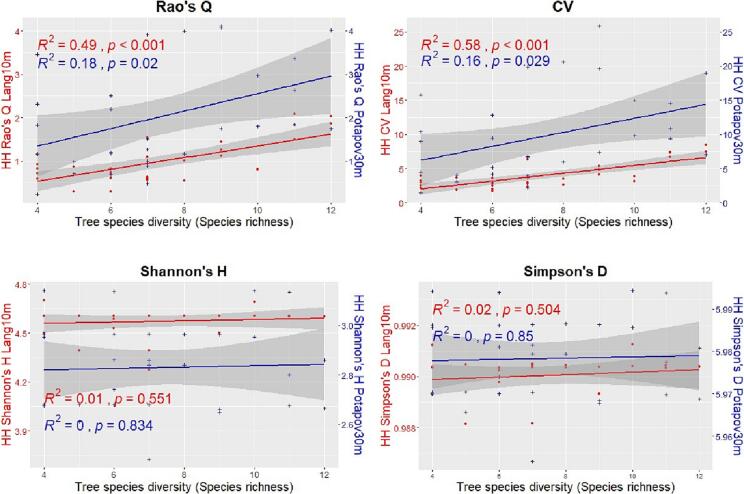
Linear regressions between the tree species diversity assessed through the species richness and the HH calculated with the 4 heterogeneity indices (Rao’s Q, CV, Shannon’s H and Simpson’s D) using both the GEDI CHMs Potapov30m (blue points and line) and Lang10m (red points and line) in the Italian study area. In order to better compare the results, in the sub-plots of Shannon’s H and the Simpson’s D, 2 different y scales have been used.

Fig. 7, Fig. 8 show, for the Italian study area, the relationships between the HH (calculated with the 4 heterogeneity indices) using both the GEDI CHMs Potapov30m and Lang10m and the tree species diversity assessed through the Shannon’s H index and the species richness respectively. In both the figures, when the HH is assessed using the GEDI CHM Lang10m, the correlations are all positive and significant (expect for the Shannon’s H and Simpson’s D heterogeneity indices when the species diversity is assessed through the species richness). Highest $R^{2}$ values are found for the Rao’s Q and CV indices ($R^{2}$=0.49 and $R^{2}$=0.58 respectively when the tree species diversity is assessed with the Shannon’s H and $R^{2}$=0.49 and $R^{2}$=0.58 respectively with the species richness). For the GEDI CHM Potapov30m the goodness of fit for each heterogeneity indices is lower than the ones of the GEDI CHM Lang10m, the correlations are all positive and significant only for the Rao’s Q and CV indices.

**Fig. 9:**
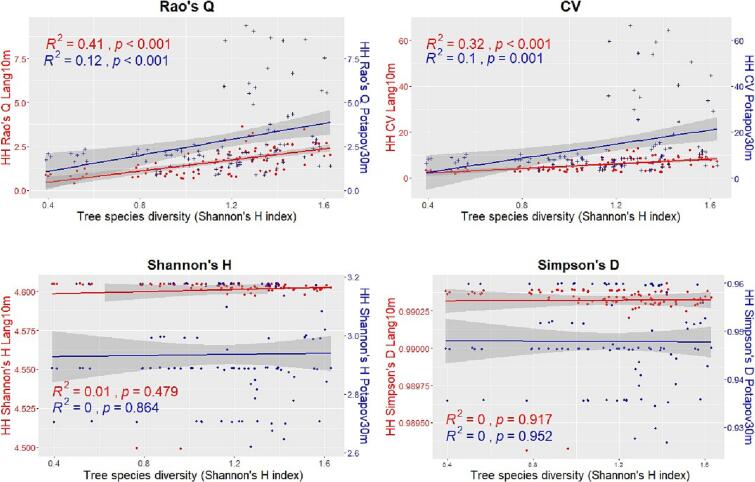
Linear regressions between the tree species diversity estimated through the Shannon’s H index and the HH calculated with the 4 heterogeneity indices (Rao’s Q, CV, Shannon’s H and Simpson’s D) using both the GEDI CHMs Potapov30m (blue points and line) and Lang10m (red points and line) in the German study area. In order to better compare the results, in the sub-plots of Shannon’s H and the Simpson’s D, 2 different y scales have been used.

**Fig. 10:**
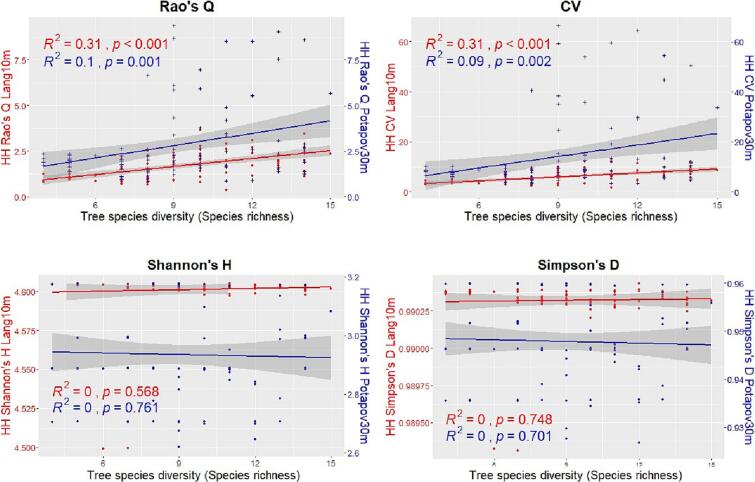
Linear regressions between the tree species diversity assessed through the species richness and the HH calculated with the 4 heterogeneity indices (Rao’s Q, CV, Shannon’s H and Simpson’s D) using both the GEDI CHMs Potapov30m (blue points and line) and Lang10m (red points and line) in the German study area.

Fig. 9, Fig. 10 show, for the German study area, the relationships between the HH (calculated with the 4 heterogeneity indices) using both the GEDI CHMs Potapov30m and Lang10m and the tree species diversity assessed through the Shannon’s H index and the species richness respectively. In both the figures, when the HH is assessed using the GEDI CHM Lang10m, the correlations are all positive and significant only for the Rao’s Q and CV. Highest $R^{2}$ values are found for the latter indices ($R^{2}$=0.41 and $R^{2}$=0.32 respectively when the tree species diversity is assessed with the Shannon’s H and $R^{2}$=0.31 for both the heterogeneity indices with the species richness). For the GEDI CHM Potapov30m the goodness of fit for each heterogeneity indices is lower than the ones of the GEDI CHM Lang10m, the correlations are positive (expect for the Simpson’s D and for Shannon’s H when the species diversity is assessed through the species richness) and significant only for the Rao’s Q and CV indices.

**Fig. 11:**
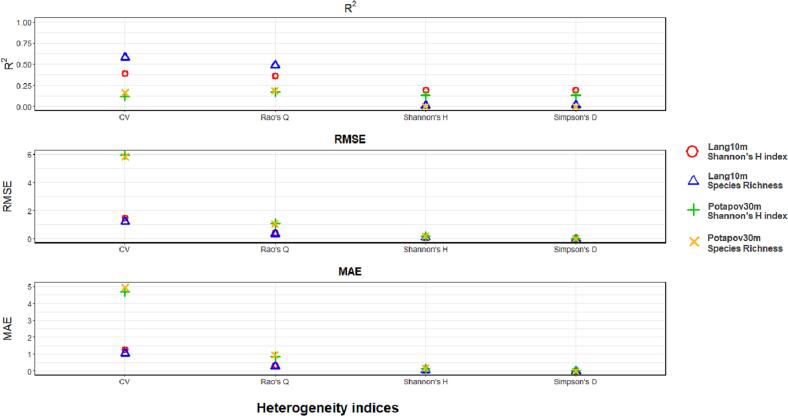
$R^{2}$, RMSE and MAE derived from the correlations between HH (calculated with the four heterogeneity indices: Rao’s Q, CV, Shannon’s H and Simpson’s D using the CHMs Lang10m and Potapov30m) and tree species diversity (assessed through the Shannon’s H index and the species richness) in the Italian study area.

**Fig. 12:**
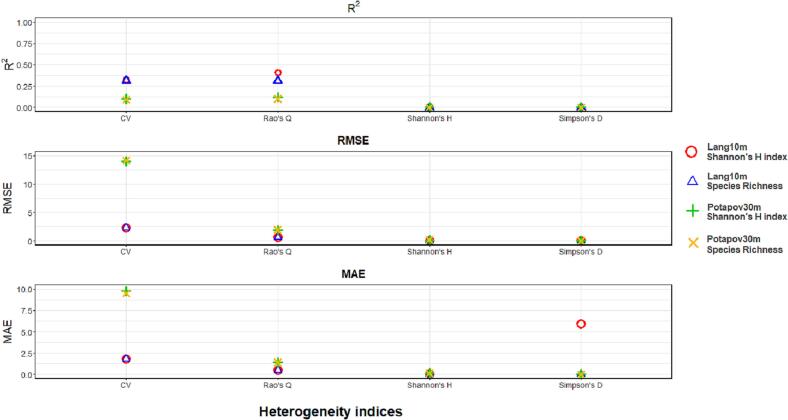
$R^{2}$, RMSE and MAE of the correlations between HH (calculated with the four heterogeneity indices: Rao’s Q, CV, Shannon’s H and Simpson’s D using the CHMs Lang10m and Potapov30m) and tree species diversity (assessed through the Shannon’s H index and the species richness) in the German study area.

Fig. 11, Fig. 12 summarize the $R^{2}$, RMSE and MAE derived from the correlations between the HH (assessed with both the GEDI CHMs Lang10m and Potapov30m) and tree species diversity (using the Shannon’s H index and species richness) for the Italian and German study area respectively. For both study sites, focusing on the correlation having the same tree species diversity measures (red dot with green cross for the Shannon’s S index and blue triangle and yellow X for species richness), $R^{2}$ values are higher in GEDI CHM Lang10m then in Potapov30. Differently, the RMSE and MAE are higher for Potapov30m than in Lang10m. Focusing on the heterogeneity indices, the Rao’ s Q index and the CV showed generally the highest $R^{2}$, and the lowest RMSE and MAE, for both the tree diversity indices and GEDI CHMs. Finally, no particular differences are shown when HH (calculated with the various heterogeneity indices) is correlated with tree species diversity estimated with both Shannon’s H and species richness.

Since in the above mentioned correlations the Rao’s Q and CV heterogeneity indices showed the best results, in particular when tested with the GEDI CHM Lang10m we decided to test the correlations with these indices and GEDI CHM in all the 130 plots using a k-fold cross-validation analysis (k = 10, repeated 3 times). The results of the analysis, shown in Table 3, highlight that the $R^{2}$ values are similar for both the heterogeneity and species diversity indices while RMSE and MAE are higher when the HH is estimated with the CV.

**Table 3:** $R^{2}$, RMSE and MAE derived from the k-fold (n = 10 repeated 3 times) cross validation between the tree species diversity (Shannon’s H and Species richness) and the HH (assessed with the GEDI CHM Lang10m using the Rao’s Q and CV indices) over the total number of plots (30 Italians  + 100 German  = 130 plots).

	***HH Rao’s Q Lang10m***		
	$R^{2}$	*RMSE* (m)	*MAE* (m)
*Shannon’s H*	0.43	0.59	0.49
*Species Richness*	0.45	0.59	0.49

	***HH CV Lang10m***		
	$R^{2}$	*RMSE* (m)	*MAE* (m)
*Shannon’s H*	0.42	2.14	1.70
*Species Richness*	0.43	2.10	1.70

Previous studies (Torresani et al., 2020, Tamburlin et al., 2021) showed that the correlation between HH and species diversity is influenced by the canopy cover and by forest density. For this reason a multiple regression analysis including these variables (canopy cover and number of trees per plot as a proxy of forest tree density) was tested in the 130 plots through a cross-validation analysis for the assessment of tree species diversity. The results shown in Table 4, highlight that the $R^{2}$ increased explaining in average 13% more of variance. The $R^{2}$ for both the indices are similar (ranging from 0.53 when the HH was calculated in the multiple regression including canopy cover, forest density and Rao’s Q and to 0.6 when the CV was included), while the RMSE and MAE are lower for the analysis with the Rao’s Q.

**Table 4:** $R^{2}$, RMSE and MAE derived from the k-fold (n = 10 repeated 3 times) cross validation between the tree species diversity (Shannon’s H and Species richness) and the HH (assessed with the GEDI CHM Lang10m using the Rao’s Q and CV indices) over the total number of plots (30 Italians  + 100 German  = 130 plots) using a multiple regression analysis (HH  + canopy cover  + number of trees).

	***Rao’s Q*** ***+*** ***canopy cover*** ***+*** ***density***		
	$R^{2}$	*RMSE* (m)	*MAE* (m)
*Shannon’s H*	0.53	0.65	0.53
*Species Richness*	0.53	0.58	0.47

	***CV*** ***+*** ***canopy cover*** ***+*** ***density***		
	$R^{2}$	*RMSE* (m)	*MAE* (m)
*Shannon’s H*	0.59	2.13	1.75
*Species Richness*	0.60	1.97	1.68

### 3.4 Correlation heterogeneity indices

**Fig. 13:**
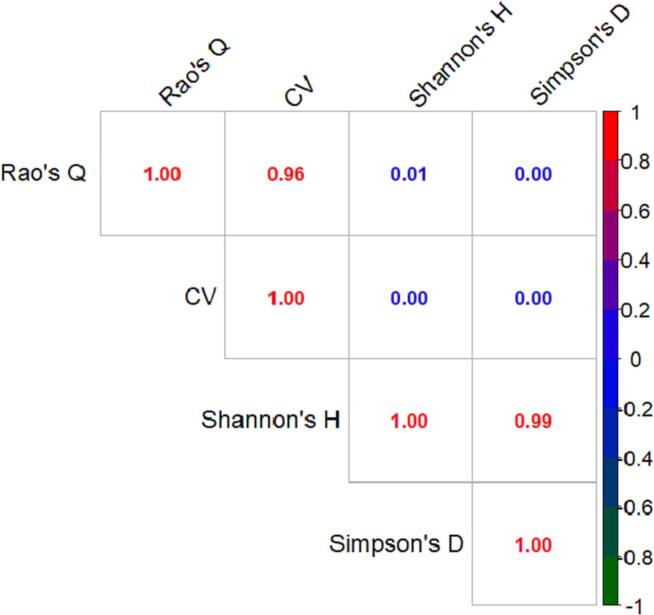
Correlation matrix ($R^{2}$) between the HH calculated with the four heterogeneity indices (Rao’s Q, CV, Shannon’s H and Simpson’s D). The data refers to the results of Table 3.

Fig. 13 shows how the HH calculated through the four different HH indices (Rao’s Q, CV, Shannon and Simpson) derived from the results of Table 3 are correlated each other by linear regression. The results shows a very strong binary picture: the HHs assessed trough Rao’s Q and CV are highly correlated while are not with Shannon’s H and Simpson’s D. On the other hand the latter are correlated with each other and not with Rao’s Q and CV.

### 3.5 Visual spatialization of the results

**Fig. 14:**
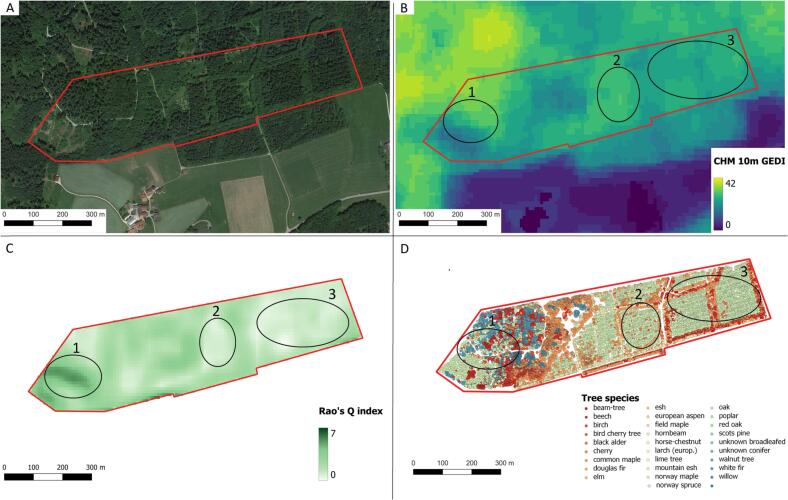
Sub-figure A shows the RGB image of the Traunstein area (Google Image June 15th 2021). Sub-figure B shows the GEDI CHM Lang10m. Sub-figure C shows the Rao’s Q values for the whole area while sub-figure D the tree species location. The circle 1 shows as an example a forested area with heterogeneous tree height (heterogeneous colours/heights in sub-figure B) with higher Rao’s Q values (dark green values in sub-figure C) and with high tree species diversity (sub-figure D). On the other hand, black circle 2 and 3 show areas with homogeneous tree height (homogeneous colours/heights in sub-figure B), low Rao’s Q values (lighter green values in sub-figure C) and low tree species richness (sub-figure D).

As an example, we decided to visually spatialize in a thematic map the correlation between the HH and tree species diversity (Fig. 14). For a practical reason we show the results over the German study area of Traunstein (one unique field and not split over different areas as the Italian plots). We calculated the HH through the Rao’s Q index with the Lang10m GEDI CHM (that according to the results of Fig. 12 reported the best results). The function “Rao” of the *rasterdiv* R package (Rocchini et al., 2021) was used to create the Rao’s Q map. We also added the map of the tree species location in order to have an overview of the tree species diversity of the area. In the figure, we highlighted as example, two distinct areas characterized by opposite values of HH and tree species diversity: the circle 1 shows a forested area with heterogeneous tree height (heterogeneous colours/heights in sub-figure B) with higher Rao’s Q values (dark green values in sub-figure C) and with high tree species diversity (sub-figure D). On the other hand, black circle 2 and 3 show areas with homogeneous tree height (homogeneous colours/heights in sub-figure B), low Rao’s Q values (lighter green values in sub-figure C) and low tree species diversity (sub-figure D).

## 4 Discussion

In this paper we tested the correlation between the *in situ* tree species diversity (assessed through the Shannon’s H index and the specie richness) and HH calculated with 4 different heterogeneity indices (Rao’s Q, CV, Shannon’s H index, Simpson’s D index) using the recently published and freely available LiDAR GEDI CHMs Lang10m (Lang et al., 2022, Lang et al., 2022) and Potapov30m (Potapov et al., 2021). We tested this correlation separately over 30 study plots situated in the Italian Alps (in the Province of Bolzano-Bozen) and in 100 plots situated in the forested area of Traunstein (Germany). Successively we tested the correlation in all the 130 plots through a cross-validation analysis including in a multiple regression, information of canopy cover and forest tree density (assessed through number of trees per plot), that in previous studies (Torresani et al., 2020, Tamburlin et al., 2021) showed to influence the correlation.

The analysis confirmed that the HH calculated from GEDI CHMs data at certain resolutions can be considered a good proxy of forest tree species diversity. The found relationship is related to the spatial complexity of the vertical forest structure: forests with high HH have a higher structural heterogeneity, with different ecological niches that can host different species (Torresani et al., 2020, Tamburlin et al., 2021). Light availability, along with other micro-climatic conditions, plays a crucial role in this relationship. Forests with a complex vertical structure let the light penetrate differently, creating different micro-habitats where both shade tolerant and intolerant species can grow (De Pauw et al., 2022, Brokaw and Scheiner, 1989).

Overall, the results showed that the correlations were good in both study areas under specific conditions (GEDI CHMs, heterogeneity and diversity indices), with slightly better $R^{2}$ values for the Italian study area characterized by higher canopy cover and number of trees. Also when tested as a whole through a cross validation analysis (Table 3) the correlations were generally strong, highlighting that the approach can hold true over different areas. As expected, the correlations improved when canopy cover and number of trees were also included in the multiple regression analysis (Table 4). In previous studies (Torresani et al., 2020, Tamburlin et al., 2021) these information have proven to play an important role in the relationship between HH and tree species diversity, showing lower performances in areas with low canopy cover, where the HH is high due to gaps in forest canopy, but tree species diversity is low. This is particularly interesting, since open areas should provide an environment where different tree species compete to share heterogeneous resources, providing numerous species niches (Schnitzer and Carson, 2001). However, it might also be expected since forest species diversity is influenced not only by the availability of light created by gaps but also by other important factors such as exposition, elevation, inclination and water/nutrient availability). For this reason we feel that further analysis have to be conducted to test this approach in different forest ecosystems and especially in areas with a low canopy cover.

Our results show that the relationship between tree species diversity and HH is influenced by a series of important factors that affect its strength and reproducibility. The first, concerns the GEDI CHM used for the assessment of HH. Generally speaking, the results showed that the HH assessed from the GEDI CHM Lang10m was better correlated to tree species diversity then when assessed with Potapov30m. This may be due the difference in spatial resolution of the two data-sets (10 m for Lang10m and 30 m for Potapov30m). According to the theory behind the HVH, the HH should reflect the forest vertical complexity, for this reason, a CHM with lower spatial resolution might be unsuitable for the estimation of tree species diversity (Torresani et al., 2020). Generally speaking, low CHM spatial resolution data make it difficult to detect smaller gaps/differences in the canopy which characterize different forest tree species and the overall biodiversity. In a previous study Torresani et al. (2020) investigated this important aspect highlighting that the finer the CHM spatial resolution for the assessment of HH, the higher the correlation with forest tree species diversity. Similarly, other studies (Huang et al., 2009, Miraki et al., 2021) showed that LiDAR data at coarse spatial resolution (or with low point density) are potential source of error in the estimation of forest vertical structure parameters (i.e. crown width and tree canopy height) and biodiversity variables.

The strongest result shown by Lang10m in comparison with the Potapov30m may be due the fact that, in both study sites, the GEDI CHM Lang10m better correlated with local ALS data then the Potapov30m (Fig. 5, Fig. 6). In the German study area the $R^{2}$ derived from the validation range from 0.73 (for Lang10m) to 0.41 (for Potapov30m) while for the Italian study area from 0.43 (for Lang10m) to 0.01 (for Potapov30m). The difference in validation accuracy between the two sites may be due to the temporal mismatch between the assessment of the GEDI CHMs (2019 and 2020 for Potapov30m and Lang10m respectively) and the ALS data acquisition (2018 and 2006 for the German and Italian study area respectively). In the Italian study area, the mismatch is relatively high (13/14 years) which makes the validating data (ALS LiDAR data) not strictly suitable to validate the GEDI CHMs (no recent LiDAR data are available). On the other hand, we are confident that in these years, there have been no serious disturbances or large clearcuts in the forest area. We feel that this is a frequent concern in studies where the temporal gap between the field data collection and LiDAR data acquisition can be significant (Polychronaki et al., 2015, Moudrỳ et al., 2021) mainly due to the infrequent LiDAR campaigns (Torresani et al., 2020). We are confident that this issue does not alter the relationships between HH and species diversity as the field data were collected in 2015 and 2017 for the German and Italian study areas, respectively. Nevertheless, the continuous acquisitions of GEDI data (April 2019 to most likely Spring 2023) will provide a rich multi-temporal data-set on various vegetation structure attributes that allow the modelling of current conditions (e.g. using Landsat 8, Sentinel-2) or previous conditions (e.g. Landsat 5, Landsat 7) (Potapov et al., 2021, Rishmawi et al., 2022). Therefore, changes in forest structure history can be characterized and related to current forest structures since those are the product of past influences (e.g. disturbances) (Potapov et al., 2021).

It is worth to underline that the accuracy of the GEDI CHMs is influenced also by other factors as the availability of the GEDI data over representative forest types and age cohorts, the optical information used to downscale the data and the presence of clouds over some areas (that can disproportionally omit some forests type while favoring others) (Potapov et al., 2021). Slopes and deep change of topography (Lefsky et al., 2007), footprint size and pulse width (Dubayah et al., 2020) together with residual geolocation uncertainties (Roy et al., 2021) are other factors that might affect the estimation of canopy height. The proprieties of the optical images and the regression algorithms used for the interpolation with GEDI data are also important factors that decrease the ability to accurately map the height of the canopy (Potapov et al., 2021, Potapov et al., 2019, Lang et al., 2019, Lang et al., 2022, Lang et al., 2022). According to these limitations GEDI LiDAR data might not be as accurate as a local (e.g. from airborne or UAV) LiDAR data (Quirós et al., 2021, Dorado-Roda et al., 2021), but they represent nonetheless an important tool used for the estimation of different forest variables such as forest biomass (Duncanson et al., 2020, Silva et al., 2021), forest growth (Guerra-Hernández and Pascual, 2021) or disturbances (Francini et al., 2022) and as shown in this study also of forest structural heterogeneity and thus of biodiversity. Most importantly, GEDI data allow to analyze and estimate these variables at global scale, over most of the Earth forest surface specially in remote areas where no local LiDAR data are available and where the vertical structure of the forest is very complex and poorly understood (Burns et al., 2020).

The field-based diversity indices and the heterogeneity measures used to assess the HH are other important factors that influenced our outcomes. Regarding the field data, our results did not show particular differences between the two used indices (Shannon’s H and species richness) reaching similar levels of correlation with the HH assessed by the same heterogeneity index. This aspect is still widely debated in the studies where the variability of remotely sensing data is used to estimate biodiversity (e.g. for the SVH). Different studies (Marzialetti et al., 2021, Rocchini et al., 2004) have shown that species richness (number of species per plot) is more strongly and sensitively correlated with the variability of the remote sensing data than the Shannon’s H index, which considers both abundance and number of species. In contrast, other studies have shown that the indices that embeds both the abundance and the richness of the considered species (e.g. the Shannon’s index) better correlate to the variability of the remote sensing data (Oldeland et al., 2010, Madonsela et al., 2017, Heumann et al., 2015). We believe that further investigations are needed to understand what drives the relationship between these different variables.

Regarding the heterogeneity indices, our results showed that the CV and the Rao’s Q index performed successfully in the assessment of the HH, in both study sites and through the cross validation analysis. The indices showed similar $R^{2}$ values but with a consistent difference in the RMSE and MAE values that were lower for the Rao’s Q index. From a theoretical point of view, the CV considers only the pixels value (through mean and standard deviation) and not their relative abundance within the plots. On the other hand, the Rao’s Q index, that has shown excellent results as spectral heterogeneity index in different SVH studies (Torresani et al., 2021, Rocchini et al., 2017, Thouverai et al., 2023), has the advantage to include both the relative abundance and the value of the pixels (through the Euclidean distance between the pixel values) (Torresani et al., 2022) thus the whole structural information derived from the LiDAR data heterogeneity. This index, when used with a single layer/raster as in this study, succeeds in becoming a good proxy of heterogeneity by narrowing to variance using half the squared Euclidean distance (1/2 $d_{\mathrm{ij}}^{2}$). We refer to (Rocchini et al., 2017, Ricotta, 2005, Ricotta and Szeidl, 2006, Ricotta et al., 2012) for further details on the mathematical characteristics of Rao’s Q. Differently, the Shannon’s H and Simpson’s D-index, used in this work, have shown to be inefficient in assessing HH. Both indices do not account for the numerical values of the GEDI CHM but they rely only on the relative abundance of the LiDAR pixels within a specific raster/area of interest (Rocchini et al., 2017). For this reason they fail to characterize the heterogeneity of tree heights that depend on both the tree height (values) and distribution (abundance).

An interesting point is related to the heterogeneity values derived from the Rao’s Q and CV from both the used GEDI CHMs. The results showed that Lang10m provide much lower values of HH than in Potapov30m (Fig. 7, Fig. 8, Fig. 9, Fig. 10). This was also found in a recent study (currently under review) (Moudrý, 2022) where the Potapov30m model, compared to Lang10m, appears to have higher CHM heterogeneity with more extreme CHM values (increasing the Rao’s Q and CV values in our study). Lang10m, having a finer spatial resolution (derived fusing GEDI data and Sentinel-2 images using a deep learning algorithm), is more spatially precise and, in our areas characterized by a generally high density (Table 2) and HH which does not reach extremely high values, properly adapted.

A major concern that might arise in this study is related to the use of the CHM for the assessment of HH thus not considering other GEDI metrics or other digital layers (e.g. optical data) related the forest structure. This choice has a twofold explanation: the first is related to the intrinsic aim of this study namely to investigate whether the recently published and freely available LiDAR GEDI CHMs developed by Lang et al., 2022, Lang et al., 2022, and by Potapov et al. (2021), could be used for estimating HH and thus tree species diversity. The second is related to the results obtained by Tamburlin et al. (2021): in their study the authors tested several LiDAR metrics (e.g., entropy and standard deviation of point cloud distribution, percentage of returns above mean height) for the estimation of HH, finding that the CHM was indeed the best metric in order to characterized the HH and tree species diversity. Similarity, Burns et al. (2020), highlighted that the metrics that most characterize the canopy structure derived from simulated GEDI LiDAR data were the most important for estimation of forest vertical variability and the spatial distribution of different species of birds. Again Fagua et al. (2021), highlighted that the LiDAR metrics that explained the variation of the impulse density namely those more sensitive to canopy stratification were the most important for prediction of alpha diversity in tropical forests. The analysis on vertical forest structure could be supplemented by relative height metrics (GEDI L2A datasets) for the characterization of low and understory vegetation. Besides the assessment of vertical forest structure, GEDI holds additional datasets on horizontal forest structure, namely total canopy cover and Plant-Area-Index, but also vertical structure complexity is derived as Foliage-Height-Diversity-Index (L2B datasets). Furthermore, novel information on above-ground biomass density (L4A dataset) could be another proxy for forest structure composition in order to delineate hot and cold spots of biodiversity (Dubayah et al., 2020).

A further concern that could emerge is related to the limited number of study areas used to test and validate our approach. As previously stressed, this approach represents a first analysis in order to assess whether the new freely available global CHMs derived by GEDI data could be used for estimating HH and thus tree species diversity. We are furthermore aware that forest biodiversity is not only affected by the tree height variability, but also by a series of factors such as light availability, topography, micro-climate and soil proprieties. Our hypothesis was not grounded on capturing directly tree species diversity but on testing an indirect method for the assessment of tree HH by GEDI CHMs LiDAR data. The outcomes highlighted that the variability of this information can be considered a proxy of the forest structure heterogeneity which in turn is related to species diversity (Torresani et al., 2020). However we are aware that the relationship between ‘high HH’ and ‘high tree species diversity’ might not always hold true in all the forests. As an example, the Swiss Stone Pine/Larch forests (*Pinus cembra/Larix decidua*), considered at the “climax state” that can be found at the limit of the vegetation in the upper altitudinal belt, are characterized by a heterogeneous vertical and horizontal structure having on the other hand a poor diversity in species; in this cold conditions, just few pioneer tree species could survive (low biodiversity) creating a structurally heterogeneous and low density forest (high HH). For this reason we consider this study as a preliminary work in understanding forest biodiversity through the use of GEDI CHM data being aware of its advantages and limitations.

## 5 Conclusion

In this paper, we examined the relationship between tree species diversity (measured by Shannon’s H index and species richness) and forest HH (assessed using four different indices) using recently published and freely available LiDAR GEDI CHMs (Lang10m and Potapov30m) in two forest areas in Europe (Italy and Germany). Our findings indicate that GEDI CHMs can be used to evaluate biodiversity patterns in forest ecosystems by estimating HH, which is related to tree species diversity. However, the results also show that the proposed method is influenced by various factors such as the GEDI CHM dataset and its related spatial resolution, the heterogeneity indices used to calculate the HH, and the forest density. Our study is a first application example but further analysis in other forest areas with different types and densities of forests, using different heterogeneity indices, are needed before the approach can be considered as a generalizable method. Additionally, it would be beneficial to also analyze the vegetation in the various herbaceous or shrub layers to have a more comprehensive view of the entire forest biodiversity. Finally, it would be valuable to combine multiple remote sensing information e.g optical (from Sentinel-2 or Landsat satellites) LiDAR (from local ALS and GEDI) or other derived products (e.g. information of topography), in order to obtain a more detailed view of heterogeneity. We suggest that this proposed approach, based on the assessment of habitat heterogeneity using recently published and freely available LiDAR GEDI CHMs, could be used by ecologists, botanists, or forest stakeholders as a preliminary analysis for identifying biodiversity hotspots, particularly in remote areas where *in situ* data are incomplete or not available and the vertical structure of the forest and its dynamics are poorly understood.

## Funding

MT and DR were partially funded by the European Union’s Horizon 2020 research and innovation program under grant agreement No 862480 (SHOWCASE). MT was also partially funded by the COST ACTION CA17134. DR was partially funded by a research project implemented under the National Recovery and Resilience Plan (NRRP), Mission 4 Component 2 Investment 1.4 - Call for tender No. 3138 of 16 December 2021, rectified by Decree n.3175 of 18 December 2021 of Italian Ministry of University and Research funded by the European Union – NextGenerationEU. Project code CN 00000033, Concession Decree No. 1034 of 17 June 2022 adopted by the Italian Ministry of University and Research, CUP J33C22001190001, Project title “National Biodiversity Future Center - NBFC”. DR was also partially funded by the Horizon Europe projects Earthbridge and B^3^.

## Declaration of Competing Interest

The authors declare that they have no known competing financial interests or personal relationships that could have appeared to influence the work reported in this paper.

## Data Availability

Data will be made available on request.

## References

[b0005] Acharya R.P., Maraseni T., Cockfield G. (2019). Global trend of forest ecosystem services valuation–an analysis of publications. Ecosyst. Serv..

[b0010] Arroyo-Rodríguez V., Fahrig L., Tabarelli M., Watling J.I., Tischendorf L., Benchimol M., Cazetta E., Faria D., Leal I.R., Melo F.P. (2020). Designing optimal human-modified landscapes for forest biodiversity conservation. Ecol. Lett..

[b0015] Betts M.G., Wolf C., Ripple W.J., Phalan B., Millers K.A., Duarte A., Butchart S.H., Levi T. (2017). Global forest loss disproportionately erodes biodiversity in intact landscapes. Nature.

[b0020] Botta-Dukát Z. (2005). Rao’s quadratic entropy as a measure of functional diversity based on multiple traits. J. Veg. Sci..

[b0025] Brokaw N.V., Scheiner S.M. (1989). Species composition in gaps and structure of a tropical forest. Ecology.

[b0030] Burns P., Clark M., Salas L., Hancock S., Leland D., Jantz P., Dubayah R., Goetz S.J. (2020). Incorporating canopy structure from simulated gedi lidar into bird species distribution models. Environ. Res. Lett..

[b0035] de Lima R.A., Oliveira A.A., Pitta G.R., de Gasper A.L., Vibrans A.C., Chave J., Ter Steege H., Prado P.I. (2020). The erosion of biodiversity and biomass in the atlantic forest biodiversity hotspot. Nat. Commun..

[b0040] De Pauw K., Sanczuk P., Meeussen C., Depauw L., De Lombaerde E., Govaert S., Vanneste T., Brunet J., Cousins S.A., Gasperini C. (2022). Forest understorey communities respond strongly to light in interaction with forest structure, but not to microclimate warming. New Phytol..

[b0045] DeJong T.M. (1975). A comparison of three diversity indices based on their components of richness and evenness. Oikos.

[b0050] Dorado-Roda I., Pascual A., Godinho S., Silva C.A., Botequim B., Rodríguez-Gonzálvez P., González-Ferreiro E., Guerra-Hernández J. (2021). Assessing the accuracy of gedi data for canopy height and aboveground biomass estimates in mediterranean forests. Remote Sens..

[b0055] Dubayah R., Armston J., Healey S.P., Bruening J.M., Patterson P.L., Kellner J.R., Duncanson L., Saarela S., Ståhl G., Yang Z. (2022). Gedi launches a new era of biomass inference from space. Environ. Res. Lett..

[b0060] Dubayah R., Blair J.B., Goetz S., Fatoyinbo L., Hansen M., Healey S., Hofton M., Hurtt G., Kellner J., Luthcke S. (2020). The global ecosystem dynamics investigation: High-resolution laser ranging of the earth’s forests and topography. Sci. Remote Sens..

[b0065] Duncanson L., Kellner J.R., Armston J., Dubayah R., Minor D.M., Hancock S., Healey S.P., Patterson P.L., Saarela S., Marselis S. (2022). Aboveground biomass density models for nasa’s global ecosystem dynamics investigation (gedi) lidar mission. Remote Sens. Environ..

[b0070] Duncanson L., Neuenschwander A., Hancock S., Thomas N., Fatoyinbo T., Simard M., Silva C.A., Armston J., Luthcke S.B., Hofton M. (2020). Biomass estimation from simulated gedi, icesat-2 and nisar across environmental gradients in sonoma county, california. Remote Sens. Environ..

[b0075] Fagua J.C., Jantz P., Burns P., Massey R., Buitrago J.Y., Saatchi S., Hakkenberg C., Goetz S.J. (2021). Mapping tree diversity in the tropical forest region of chocó-colombia. Environ. Res. Lett..

[b0080] Francini S., D’Amico G., Vangi E., Borghi C., Chirici G. (2022). Integrating gedi and landsat: spaceborne lidar and four decades of optical imagery for the analysis of forest disturbances and biomass changes in italy. Sensors.

[b0085] Gholizadeh A., Žižala D., Saberioon M., Boruvka L. (2018). Soil organic carbon and texture retrieving and mapping using proximal, airborne and sentinel-2 spectral imaging. Remote Sens. Environ..

[b0090] Guerra-Hernández J., Pascual A. (2021). Using gedi lidar data and airborne laser scanning to assess height growth dynamics in fast-growing species: a showcase in spain. For. Ecosyst..

[b0095] Gupta R., Sharma L.K. (2022). Mixed tropical forests canopy height mapping from spaceborne lidar gedi and multisensor imagery using machine learning models. Remote Sens. Appl.: Soc. Environ..

[b0100] Hakkenberg C., Zhu K., Peet R., Song C. (2018). Mapping multi-scale vascular plant richness in a forest landscape with integrated lidar and hyperspectral remote-sensing. Ecology.

[b0105] Hansen M.C., Potapov P.V., Moore R., Hancher M., Turubanova S.A., Tyukavina A., Thau D., Stehman S.V., Goetz S.J., Loveland T.R. (2013). High-resolution global maps of 21st-century forest cover change. Science.

[b0110] Heumann B.W., Hackett R.A., Monfils A.K. (2015). Testing the spectral diversity hypothesis using spectroscopy data in a simulated wetland community. Ecol. Inform..

[b0115] Hoffrén R., Lamelas M.T., de la Riva J., Domingo D., Montealegre A.L., García-Martín A., Revilla S. (2023). Assessing gedi-nasa system for forest fuels classification using machine learning techniques. Int. J. Appl. Earth Obs. Geoinf..

[b0120] Huang H., Gong P., Cheng X., Clinton N., Li Z. (2009). Improving measurement of forest structural parameters by co-registering of high resolution aerial imagery and low density lidar data. Sensors.

[b0125] Kacic P., Hirner A., Da Ponte E. (2021). Fusing sentinel-1 and-2 to model gedi-derived vegetation structure characteristics in gee for the paraguayan chaco. Remote Sens..

[b0130] Kacic P., Kuenzer C. (2022). Forest biodiversity monitoring based on remotely sensed spectral diversity—a review. Remote Sens..

[b0135] Kumar P., Dobriyal M., Kale A., Pandey A., Tomar R., Thounaojam E. (2022). Calculating forest species diversity with information-theory based indices using sentinel-2a sensor’s of mahavir swami wildlife sanctuary. Plos One.

[b0140] Lang, N., Jetz, W., Schindler, K., Wegner, J.D., 2022. A high-resolution canopy height model of the earth. arXiv preprint arXiv:2204.08322.

[b0145] Lang N., Kalischek N., Armston J., Schindler K., Dubayah R., Wegner J.D. (2022). Global canopy height regression and uncertainty estimation from gedi lidar waveforms with deep ensembles. Remote Sens. Environ..

[b0150] Lang N., Schindler K., Wegner J.D. (2019). Country-wide high-resolution vegetation height mapping with sentinel-2. Remote Sens. Environ..

[b0155] Lefsky M.A., Keller M., Pang Y., De Camargo P.B., Hunter M.O. (2007). Revised method for forest canopy height estimation from geoscience laser altimeter system waveforms. J. Appl. Remote Sens..

[b0160] Levin N., Shmida A., Levanoni O., Tamari H., Kark S. (2007). Predicting mountain plant richness and rarity from space using satellite-derived vegetation indices. Divers. Distrib..

[b0165] Liu X., Su Y., Hu T., Yang Q., Liu B., Deng Y., Tang H., Tang Z., Fang J., Guo Q. (2022). Neural network guided interpolation for mapping canopy height of china’s forests by integrating gedi and icesat-2 data. Remote Sens. Environ..

[b0170] Madonsela S., Cho M.A., Ramoelo A., Mutanga O. (2017). Remote sensing of species diversity using landsat 8 spectral variables. ISPRS J. Photogramm. Remote Sens..

[b0175] Marselis S.M., Keil P., Chase J.M., Dubayah R. (2022). The use of gedi canopy structure for explaining variation in tree species richness in natural forests. Environ. Res. Lett..

[b0180] Marzialetti F., Cascone S., Frate L., Di Febbraro M., Acosta A.T.R., Carranza M.L. (2021). Measuring alpha and beta diversity by field and remote-sensing data: A challenge for coastal dunes biodiversity monitoring. Remote Sens..

[b0185] Marzialetti F., Di Febbraro M., Malavasi M., Giulio S., Acosta A.T.R., Carranza M.L. (2020). Mapping coastal dune landscape through spectral rao’s q temporal diversity. Remote Sens..

[b0190] Michele T., Duccio R., Marc Z., Ruth S., Giustino T. (2018). IGARSS 2018–2018 IEEE International Geoscience and Remote Sensing Symposium.

[b0195] Miraki M., Sohrabi H., Fatehi P., Kneubuehler M. (2021). Individual tree crown delineation from high-resolution uav images in broadleaf forest. Ecol. Inform..

[b0200] Mittermeier R.A., Myers N., Thomsen J.B., Da Fonseca G.A., Olivieri S. (1998). Biodiversity hotspots and major tropical wilderness areas: approaches to setting conservation priorities. Conserv. Biol..

[b0205] Moudrỳ V., Cord A.F., Gábor L., Laurin G.V., Barták V., Gdulová K., Malavasi M., Rocchini D., Stereńczak K., Prošek J. (2023). Vegetation structure derived from airborne laser scanning to assess species distribution and habitat suitability: The way forward. Divers. Distrib..

[b0210] Moudrỳ V., Moudrá L., Barták V., Bejček V., Gdulová K., Hendrychová M., Moravec D., Musil P., Rocchini D., Št’astnỳ K. (2021). The role of the vegetation structure, primary productivity and senescence derived from airborne lidar and hyperspectral data for birds diversity and rarity on a restored site. Landsc. Urban Plann..

[b0215] Moudrý, V., 2022. Accuracy of recent global canopy height maps and their applicability for biodiversity modelling in temperate biomes.

[b0220] Oldeland J., Wesuls D., Rocchini D., Schmidt M., Jürgens N. (2010). Does using species abundance data improve estimates of species diversity from remotely sensed spectral heterogeneity?. Ecol. Ind..

[b0225] Palmer M.W., Earls P.G., Hoagland B.W., White P.S., Wohlgemuth T. (2002). Quantitative tools for perfecting species lists. Environ.: Offic. J. Int. Environ. Soc..

[b0230] Pan Y., Birdsey R.A., Phillips O.L., Jackson R.B. (2013). The structure, distribution, and biomass of the world’s forests. Annu. Rev. Ecol. Evol. Syst..

[b0235] Polychronaki A., Spindler N., Schmidt A., Stoinschek B., Zebisch M., Renner K., Sonnenschein R., Notarnicola C. (2015). Integrating rapideye and ancillary data to map alpine habitats in south tyrol, italy. Int. J. Appl. Earth Obs. Geoinf..

[b0240] Potapov P., Li X., Hernandez-Serna A., Tyukavina A., Hansen M.C., Kommareddy A., Pickens A., Turubanova S., Tang H., Silva C.E. (2021). Mapping global forest canopy height through integration of gedi and landsat data. Remote Sens. Environ..

[b0245] Potapov P., Tyukavina A., Turubanova S., Talero Y., Hernandez-Serna A., Hansen M., Saah D., Tenneson K., Poortinga A., Aekakkararungroj A. (2019). Annual continuous fields of woody vegetation structure in the lower mekong region from 2000–2017 landsat time-series. Remote Sens. Environ..

[b0250] Primack R.B. (2006).

[b0255] Quirós E., Polo M.-E., Fragoso-Campón L. (2021). Gedi elevation accuracy assessment: a case study of southwest spain. IEEE J. Select. Top. Appl. Earth Obs. Remote Sens..

[b0260] Rao C.R. (1982). Diversity and dissimilarity coefficients: a unified approach. Theor. Popul. Biol..

[b0265] Ricotta C. (2005). Additive partitioning of rao’s quadratic diversity: a hierarchical approach. Ecol. Model..

[b0270] Ricotta C., Pavoine S., Bacaro G., Acosta A.T. (2012). Functional rarefaction for species abundance data. Methods Ecol. Evol..

[b0275] Ricotta C., Szeidl L. (2006). Towards a unifying approach to diversity measures: bridging the gap between the shannon entropy and rao’s quadratic index. Theor. Popul. Biol..

[b0280] Rishmawi K., Huang C., Schleeweis K., Zhan X. (2022). Integration of viirs observations with gedi-lidar measurements to monitor forest structure dynamics from 2013 to 2020 across the conterminous united states. Remote Sens..

[b0285] Rishmawi K., Huang C., Zhan X. (2021). Monitoring key forest structure attributes across the conterminous united states by integrating gedi lidar measurements and viirs data. Remote Sens..

[b0290] Rocchini D. (2007). Effects of spatial and spectral resolution in estimating ecosystem *α*)diversity by satellite imagery. Remote Sens. Environ..

[b0295] Rocchini D., Chiarucci A., Loiselle S.A. (2004). Testing the spectral variation hypothesis by using satellite multispectral images. Acta Oecol..

[b0300] Rocchini D., Marcantonio M., Ricotta C. (2017). Measuring rao’s q diversity index from remote sensing: An open source solution. Ecol. Ind..

[b0305] Rocchini D., Salvatori N., Beierkuhnlein C., Chiarucci A., De Boissieu F., Förster M., Garzon-Lopez C.X., Gillespie T.W., Hauffe H.C., He K.S. (2021). From local spectral species to global spectral communities: A benchmark for ecosystem diversity estimate by remote sensing. Ecol. Inform..

[b0310] Rocchini D., Santos M.J., Ustin S.L., Féret J.-B., Asner G.P., Beierkuhnlein C., Dalponte M., Feilhauer H., Foody G.M., Geller G.N. (2022). The spectral species concept in living color. Journal of Geophysical Research. Biogeosciences.

[b0315] Rocchini D., Thouverai E., Marcantonio M., Iannacito M., Da Re D., Torresani M., Bacaro G., Bazzichetto M., Bernardi A., Foody G.M. (2021). rasterdiv—an information theory tailored r package for measuring ecosystem heterogeneity from space: To the origin and back. Methods Ecol. Evol..

[b0320] Rocchini D., Torresani M., Beierkuhnlein C., Feoli E., Foody G.M., Lenoir J., Malavasi M., Moudrỳ V., Šímová P., Ricotta C. (2022). Double down on remote sensing for biodiversity estimation: a biological mindset. Commun. Ecol..

[b0325] Roy D.P., Kashongwe H.B., Armston J. (2021). The impact of geolocation uncertainty on gedi tropical forest canopy height estimation and change monitoring. Sci. Remote Sens..

[b0330] Saarela S., Holm S., Healey S.P., Patterson P.L., Yang Z., Andersen H.-E., Dubayah R.O., Qi W., Duncanson L.I., Armston J.D. (2022). Comparing frameworks for biomass prediction for the global ecosystem dynamics investigation. Remote Sens. Environ..

[b0335] Schnitzer S.A., Carson W.P. (2001). Treefall gaps and the maintenance of species diversity in a tropical forest. Ecology.

[b0340] Shannon C.E. (1948). A mathematical theory of communication. Bell Syst. Tech. J..

[b0345] Silva C.A., Duncanson L., Hancock S., Neuenschwander A., Thomas N., Hofton M., Fatoyinbo L., Simard M., Marshak C.Z., Armston J. (2021). Fusing simulated gedi, icesat-2 and nisar data for regional aboveground biomass mapping. Remote Sens. Environ..

[b0350] Skidmore A.K., Coops N.C., Neinavaz E., Ali A., Schaepman M.E., Paganini M., Kissling W.D., Vihervaara P., Darvishzadeh R., Feilhauer H. (2021). Priority list of biodiversity metrics to observe from space. Nat. Ecol. Evol..

[b0355] Sun H., Hu J., Wang J., Zhou J., Lv L., Nie J. (2021). Rspd: A novel remote sensing index of plant biodiversity combining spectral variation hypothesis and productivity hypothesis. Remote Sens..

[b0360] Tagliabue G., Panigada C., Celesti M., Cogliati S., Colombo R., Migliavacca M., Rascher U., Rocchini D., Schüttemeyer D., Rossini M. (2020). Sun–induced fluorescence heterogeneity as a measure of functional diversity. Remote Sens. Environ..

[b0365] Tamburlin D., Torresani M., Tomelleri E., Tonon G., Rocchini D. (2021). Testing the height variation hypothesis with the r rasterdiv package for tree species diversity estimation. Remote Sens..

[b0370] Thouverai E., Marcantonio M., Lenoir J., Galfré M., Marchetto E., Bacaro G., Gatti R.C., Da Re D., Di Musciano M., Furrer R. (2023). Integrals of life: Tracking ecosystem spatial heterogeneity from space through the area under the curve of the parametric rao’s q index. Ecol. Complex..

[b0375] Torresani M., Feilhauer H., Rocchini D., Féret J.-B., Zebisch M., Tonon G. (2021). Which optical traits enable an estimation of tree species diversity based on the spectral variation hypothesis?. Appl. Veg. Sci..

[b0380] Torresani M., Masiello G., Vendrame N., Gerosa G., Falocchi M., Tomelleri E., Serio C., Rocchini D., Zardi D. (2022). Correlation analysis of evapotranspiration, emissivity contrast and water deficit indices: A case study in four eddy covariance sites in italy with different environmental habitats. Land.

[b0385] Torresani M., Rocchini D., Sonnenschein R., Zebisch M., Hauffe H.C., Heym M., Pretzsch H., Tonon G. (2020). Height variation hypothesis: A new approach for estimating forest species diversity with chm lidar data. Ecol. Ind..

[b0390] Torresani M., Rocchini D., Sonnenschein R., Zebisch M., Marcantonio M., Ricotta C., Tonon G. (2019). Estimating tree species diversity from space in an alpine conifer forest: The rao’s q diversity index meets the spectral variation hypothesis. Ecol. Inform..

[b0395] White J.C., Gómez C., Wulder M.A., Coops N.C. (2010). Characterizing temperate forest structural and spectral diversity with hyperion eo-1 data. Remote Sens. Environ..

